# Sterol and lipid analyses identifies hypolipidemia and apolipoprotein disorders in autism associated with adaptive functioning deficits

**DOI:** 10.1038/s41398-021-01580-8

**Published:** 2021-09-09

**Authors:** Elaine Tierney, Alan T. Remaley, Audrey Thurm, Leah R. Jager, Christopher A. Wassif, Lisa E. Kratz, Joan E. Bailey-Wilson, Irena Bukelis, Geeta Sarphare, Eun Sol Jung, Boudewien Brand, Kelly K. Noah, Forbes D. Porter

**Affiliations:** 1 Kennedy Krieger Institute, 707N. Broadway, Room 400J, Baltimore, MD USA; 2 Department of Psychiatry and Behavioral Sciences, Johns Hopkins University School of Medicine, Baltimore, MD USA; 3 Cardiovascular and Pulmonary Branch, National Heart, Lung, and Blood Institute, Bethesda, MD USA; 4 Neurodevelopmental and Behavioral Phenotyping Service, Office of the Clinical Director, National Institute of Mental Health, Bethesda, MD USA; 5 Department of Biostatistics, Johns Hopkins Bloomberg School of Public Health, Baltimore, MD USA; 6 Division of Intramural Research, Eunice Kennedy Shriver National Institute of Child Health and Human Development, Bethesda, MD USA; 7 Computational Statistical Genomics Branch, National Human Genome Research Institute Baltimore, Baltimore, MD USA; 8 Present Address: Department of Psychiatry, University of Alabama at Birmingham, Birmingham, AL USA; 9 Present Address: Therapeutic Superventions, Inc., 14373 Howard Road, Dayton, MD 21036 USA

**Keywords:** Predictive markers, Autism spectrum disorders

## Abstract

An improved understanding of sterol and lipid abnormalities in individuals with autism spectrum disorder (ASD) could lead to personalized treatment approaches. Toward this end, in blood, we identified reduced synthesis of cholesterol in families with ≥2 children with ASD participating with the Autism Genetic Resource Exchange (AGRE), as well as reduced amounts of high-density lipoprotein cholesterol (HDL), apolipoprotein A1 (ApoA1) and apolipoprotein B (ApoB), with 19.9% of the subjects presenting with apolipoprotein patterns similar to hypolipidemic clinical syndromes and 30% with either or both ApoA1 and ApoB less than the fifth centile. Subjects with levels less than the fifth centile of HDL or ApoA1 or ApoA1 + ApoB had lower adaptive functioning than other individuals with ASD, and hypocholesterolemic subjects had apolipoprotein deficits significantly divergent from either typically developing individuals participating in National Institutes of Health or the National Health and Nutrition Examination Survey III.

## Introduction

Autism spectrum disorder (ASD) is the unitary diagnostic classification for a heterogeneous neurodevelopmental disorder for which underlying etiologies have not been identified in most cases. The classification of ASD presently encompasses deficits in social-emotional reciprocity, nonverbal communicative behavior used for social interaction, and relationships and by the presence of restricted, repetitive patterns of behavior, or interests [1]. In this study, we examine sterol levels in ASD, for which cholesterol and desmosterol play essential roles during embryogenesis, myelination, and brain development [2–4]. In the periphery, high-density lipoprotein cholesterol (HDL) and low-density lipoprotein cholesterol (LDL) are the main lipoproteins responsible for cholesterol transport, with their primary structural proteins apolipoprotein A1 (ApoA1) in HDL and apolipoprotein B (ApoB) in LDL. HDL also transports proteins, hormones, carotenoids, vitamins, bioactive lipids, and 90% of extracellular micro-ribonucleic acids (miRNA), and a reduction in HDL levels leads to reduced miRNA and changes in gene expression [5]. Dysregulated miRNA has been implicated in ASD [6–8]. The adrenal glands obtain cholesterol from HDL and LDL to synthesize steroid hormones and neuroactive steroids [9, 10]. In the brain, the latter promote dendrite growth and synaptogenesis, exhibit neuroprotective and neurodevelopmental effects, and modulate neurotransmitter receptor activity. Decreased neuroactive steroid levels [11] and sterol-related abnormal levels of cortisol, testosterone, estrogen, progesterone, and vitamin D have been reported in ASD [12–14].

Cholesterol does not cross from the periphery into the central nervous system (CNS) and small HDL particles possibly traverse by endocytosis to a small extent [15]. Therefore, cholesterol, HDL-like particles, and apolipoprotein E (ApoE) are synthesized in situ by glial cells [16–18]. However, all ApoA1 in the CNS traverses from the periphery [19]. Cholesterol reduction in neurons impairs synaptic vesicle exocytosis, neuronal activity, and neurotransmission and leads to dendritic spine and synapse degradation [20]. Altered cholesterol, HDL and LDL metabolism, gene variants of *APOA1*, *APOB*, and *APOE4*, and decreased ApoA1 levels have been implicated in other neurologic disorders [21–35]. Mutations in genes that alter synaptic function can also lead to intellectual disability and ASD [36]. Mammalian target of rapamycin pathway is affected by cholesterol homeostatic changes [37] and is dysregulated in individuals with phosphatase and tensin homolog mutations, tuberous sclerosis, neurofibromatosis, fragile X syndrome, and Rett syndrome [38]. Individuals with fragile X syndrome have low cholesterol, HDL, LDL, and ApoA1 levels [39–41], and their degree of maladaptive behaviors occurs in inverse relationship to cholesterol levels [40]. Individuals with Rett syndrome and the related CDKL5 disorder have elevated lipid levels [42, 43].

Smith–Lemli–Opitz syndrome (SLOS) is caused by the deficiency of 7-dehydrocholesterol (7DHC) reductase, the enzyme responsible for the last step of cholesterol biosynthesis [44], resulting from mutations in *DHCR7* [45–47]. The SLOS anatomical severity score and gross neurologic pathology inversely correlate with cholesterol and the ratio of 7DHC + 8-dehydrocholesterol (8DHC) to cholesterol [48, 49]; the occipital frontal head circumference (OFC) is proportionately the smallest measurement at birth and is 2 standard deviations (SD) below the typical population [50, 51]. ASD is frequently observed in SLOS [52, 53]; however, the rate of ASD clinical diagnosis appears to be artificially increased due to lower cognitive function. Serum cholesterol, serum 7DHC and cerebral spinal fluid 7DHC negatively correlate with intelligence (intelligence quotient (IQ)) and adaptive functioning (life skills) [49]. Individuals with severe SLOS have reduced levels of alpha-apolipoprotein (defined as ApoA1 and ApoA without subclassification) than individuals with mild SLOS and significantly less alpha-apolipoprotein than an age-matched typical control group. Levels of ApoB did not differ between these two SLOS groups and typical controls [54, 55].

Studies of lipid dysregulation using ASD spectrum blood samples include findings of low cholesterol and lathosterol levels [56]; low cholesterol levels with lower IQ, higher anxiety, and depression [57]; low HDL and elevated triglyceride levels [58]; low cholesterol and very-low-density lipoprotein levels [59]; reduced ApoB precursor peptides with higher ApoA1 and ApoB protein precursor levels in ASD with higher IQ [60]; increased lipoprotein lipase activity [61]; dysregulation of lipid transport and metabolism proteins [62]; enrichment of lipid metabolism pathways [63]; increased cholesterol levels with Asperger syndrome [64]; and identification of exons regulating both neurodevelopment and fat metabolism with an increased rate of dyslipidemia diagnoses [65].

**Fig. 1:**
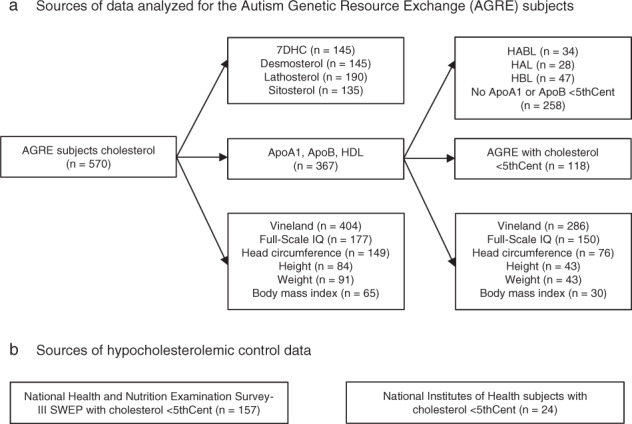
AGRE and control subject samples and data analyzed. **a** Cholesterol (CHL) analyses (*n* = 570) were performed. HDL, apolipoprotein A1 (ApoA1), and ApoB (each *n* = 367) analyses were performed in a subset of subjects. AGRE Vineland Scales of Adaptive Behavior, IQ, and physical parameter subsets were analyzed. ApoA1 and ApoB values less than the 5th centile (<5thCent) and greater than or equal to the 5th centile (≥5thCent) were organized into patterns: HABL (ApoA1 < 5thCent and ApoB < 5thCent); HAL (ApoA1 < 5thCent and ApoB ≥ 5thCent); HBL (ApoA1 ≥ 5thCent and ApoB < 5thCent); Normal (ApoA1 ≥ 5thCent and ApoB ≥ 5thCent). The 118 CHL < 5thCent subjects with apolipoprotein testing, age 4.0–17.9 years, were analyzed with CHL < 5thCent control data. **b** ApoA1 and ApoB analyses were performed with blood samples from National Institutes of Health (NIH) typically developing control subjects and National Health and Nutrition Examination Survey III survey-weighted estimates of the percentage in the USA typical population (NHANES-III-SWEP), age 4.0–17.9 years.

Herein we measured blood levels of 7DHC, lathosterol, desmosterol, sitosterol, cholesterol, HDL, ApoA1, and ApoB in individuals with ASD who participated in the Autism Genetic Resource Exchange (AGRE) and assigned age- and sex-specific centiles generated from the population-survey National Health and Nutrition Examination Survey III (NHANES-III) [66] to their cholesterol, HDL, ApoA1, and ApoB levels. These lipid values were analyzed with pre-existing AGRE data sets of measures of adaptive functioning, IQ, physical parameters, and medical history (Fig. 1). We analyzed the differences in levels of 7DHC, lathosterol, desmosterol, sitosterol, adaptive functioning, and IQ between groups of AGRE subjects with cholesterol, HDL, ApoA1, and ApoB levels less than the fifth centile (<5thCent) and greater than or equal to the 5th centile (≥5thCent) (<5thCent&≥5thCent-groups). We determined the number of individuals with patterns of cholesterol, HDL, ApoA1, and ApoB that approximated hypocholesterolemic clinical disorders. We also tested for differences in adaptive function and IQ by patterns of low ApoA1 and ApoB levels and compared the ApoA1 and ApoB pattern in hypocholesterolemic (<5thCent) AGRE subjects, typically developing subjects, and NHANES-III individuals.

## Materials/subjects and methods

We performed the study in compliance with the Code of Ethics of the World Medical Association (Declaration of Helsinki) and standards established by the Institutional Review Boards of AGRE, Johns Hopkins Medicine, and the National Institutes of Health (NIH). Informed consent was not required to use the NHANES-III data, which are de-identified and available for download at the Centers for Disease Control (CDC)’s website. Informed consent was obtained from all other participants, with the consent process performed by investigators of AGRE or the NIH.

There were three sources of data.

### (1) Autism Genetics Research Exchange (AGRE) subjects’ serum or plasma samples and phenotypic data

AGRE participants in our study were collected by the AGRE consortium [67, 68] and AGRE had the following inclusion criteria: (1) the family had ≥2 children diagnosed with autism, pervasive developmental disorder-not otherwise specified, or Asperger syndrome by a physician or autism specialist before contacting AGRE, (2) the child had not participated in any other autism gene repository, (3) once enrolled with AGRE, the subject met the AGRE diagnostic criteria of either autism, “not quite autism,” or “broad spectrum.”

Our AGRE subject inclusion criteria were (1) a diagnosis of autism, not quite autism or broad spectrum assigned by AGRE, (2) ≥2 children with autism, not quite autism or broad spectrum in the same sibship (a multiplex family), (3) any age, (4) no fragile X gene pre-mutation or full mutation in either the subject or sibling, (5) no genetic disorder other than trisomy 21 in the subject or sibling, (6) enrollment of only one individual per family (independent subjects). We included all AGRE subjects who met the inclusion criteria and for whom there were samples available in the AGRE repository.

From AGRE, we obtained frozen serum or plasma samples, gathered by AGRE from 570 subjects between the years of 1997 to 2012 (year mean ± SD: 2004.4 ± 5.1). From AGRE, we obtained all available information regarding pedigree, medical history, fragile X testing, karyotype analyses, physical measurements, and medications taken at the time of evaluation, as well as behavioral measures of ASD diagnosis, adaptive function, and intelligence/development. The 570 subjects’ ages ranged from 1.9 to 33.5 years (471 males, 99 females), with 47 subjects <4.0 years, (41 males, 6 females) and 27 subjects >17.9 years (24 males, 3 females). Fifty-six were from triplet or twin sets who had zygosity testing confirmed by AGRE: 7 from triplet sets (all dizygotic), 49 from twin sets (44 dizygotic and 5 monozygotic). The subjects from 4 of the monozygotic sets had both the twin and another sibling who had autism, not quite autism, or broad spectrum. The remaining monozygotic twin had the twin and a first cousin with autism. The ancestry information was communicated by the parent to AGRE and this data was used to generate the following percentages for “race”: American Indian or Alaskan Native (0.2%), Native Hawaiian or other Pacific Islander (0.9%), Asian (2.1%), Black or African American (2.6%), More than one race (4.0%), Unknown (4.6%), and White (85.6%). The self-reported percentages for “ethnicity” were Unknown (5.1%), Hispanic or Latino (13.2%), and Not Hispanic or Latino (81.7%).

AGRE arranged for fragile X gene testing in a Clinical Laboratory Improvement Amendment (CLIA)-certified laboratory for 407 subjects, and, for the remaining subjects, AGRE had fragile X testing performed for a sibling with either autism, not quite autism, or broad spectrum. No subject or sibling had a fragile X pre-mutation or full mutation. Of the 570 subjects, 158 had a normal karyotype examination with G banding, 79 had SNRPN, and 149 had telomere examination. An additional 12 participants were reported by their parents as having a normal karyotype except for 1 subject with trisomy 21, and that subject had a sibling with autism. We obtained medical history interviews for 150 subjects and 66 were on no medication, 76 were on medication, and 8 did not supply information as to the medication status. For the individuals who were on medication, only 5 completed the medical history form in the same year of age as the blood was collected. The mean difference in years between the ages that the blood samples were drawn and the medical history interviews were performed was 2.2 ± 1.5 years. Of the 76 on medication at the time of the completion of the medical history interview, 61 reported at least one medication that could affect appetite or cause lipid dysregulation: 11 on stimulants (which could decrease appetite); 14 on antiepileptics (which could increase appetite); 22 on atypical neuroleptics (which could increase appetite and increase lipid levels); 28 on antidepressants (which could increase or decrease appetite); 1 on a statin (which could decrease lipid levels). No subject was on a diabetes medication.

#### Measurements of adaptive functioning and intelligence

From AGRE, we obtained Vineland Adaptive Behavior Scales (Vineland) [69] data for 404 subjects. The Vineland is an interview administered to the caretaker to assess abilities typically observed from birth to 19 years. Raw scores from the Communication, Daily Living Skills, Socialization, and Motor Skills domains were converted to standard scores (SS). The Vineland Adaptive Behavior Composite (Vineland-ABC) SS includes the domains noted above and reflects overall adaptive ability (all SS have a mean of 100 and SD of 15). SS <70 are considered impaired, 70–79 borderline, 80–89 below average, and 90+ average.

From AGRE, we obtained Stanford-Binet Intelligence Scales, 5th edition (SB) [70] (*n* = 164) and Mullen Scales of Early Learning (Mullen) [71] (*n* = 13) data that we used to determine intelligence/developmental functioning. AGRE reported full-scale intelligence quotient (FSIQ), nonverbal IQ, and verbal IQ for the SB. For comparability (and to accommodate out-of-age-range testing), we calculated corresponding scores from the Mullen to derive developmental quotients (DQ). We calculated the Mullen nonverbal DQ (NVDQ) from the nonverbal mental age (by averaging the Visual Reception and the Fine Motor Domains scores in mental age) and divided that value by chronological age. We calculated the Mullen verbal DQ (VDQ) from the verbal mental age (by averaging the Expressive and Receptive Language Domains in mental age) and divided that value by chronological age. We averaged the NVDQ and VDQ to derive the Mullen full-scale DQ. Hereafter, we use the term IQ to refer to both IQ and DQ scores. We averaged the SB and Mullen FSIQs to create FSIQ. IQ scores have a mean of 100 and a SD of 15; by convention, IQ scores are described using the following categories: ≥85 average, 84–70 borderline, 69–55 mild impairment, 54–40 moderate impairment, 39–25 severe impairment, and <25 profound impairment.

### (2) National Health and Nutrition Survey III (NHANES-III) [66] data for the creation of age-and-sex-specific lipid centiles

The CDC NHANES-III was a nationwide probability sample of 39,695 persons that was conducted from 1988 to 994. The NHANES-III pediatric cholesterol [72, 73] and ApoA1 and ApoB [74] data were previously analyzed and the authors demonstrated that lipid levels vary by age and sex throughout childhood and adolescence. Analysis of data from the NHANES 1999–2006 and Project HeartBeat! [75] (two independent but contemporaneous studies), as well as the Lipid Research Clinics Program Prevalence Study [76], confirmed that serum total cholesterol concentration over the age range from 8 through 17 years varies significantly in relation to age and sex. Data from NHANES 1999–2004 indicated that mean levels of total cholesterol for children and adolescents (4–19 years old) was 1 mg/dL lower since NHANES-III (1988–1994) and there was little change in mean concentrations of LDL-cholesterol [76]. The previously published age- and sex-specific lipid centile tables list the values in 2–7 year increments of age and in 5–25 centile increments [72–74, 77], but we sought to have greater precision in the assignment of centiles. Therefore, using survey-weighting methods, we used all available fasting and non-fasting NHANES-III subject data to generate age- and sex-specific centile tables utilizing the age at the exam (NHANES-III variable MXPAXTMR) and the sex (variable HSSEX) as well as the NHANES-III variables for cholesterol (TCP), HDL (HDP), ApoA1 (AAP), and ApoB (ABP) levels for all ages available (≥4 years) in increments of 2–3 years of age and 1 centile. To create the pediatric calculated LDL levels, we used the Friedewald formula for those who had fasted for at least 9 h (using variable PHPFAST) and had triglycerides levels (variable TGP) ≤400: LDL = total cholesterol − HDL − triglycerides/5. To create the adult calculated LDL table, the variable LCP was used. However, because the fasting status of the subjects was not known, the calculated LDL and triglyceride tables were not used in the analyses (Supplementary Tables 1–12).

### (3) NIH typically developing low cholesterol comparison group

We performed apolipoprotein analyses on serum or plasma samples previously collected from all available subjects of a NIH typically developing comparison group who were identified as having cholesterol <5thCent while serving as a control population in an NIH study that was not related to lipid disorders and were between the ages of 4.0 and 17.9 years. The NIH original study’s subject inclusion criteria were (1) no history of developmental delay, (2) FSIQ scores no more than 1.5 SD below the mean, (3) no family member with an autism spectrum diagnosis, (4) testing with the Autism Diagnostic Interview-R, Autism Diagnostic Observation Schedule [78] and clinical testing performed at NIH revealed no clinical concerns regarding an ASD diagnosis [79, 80]. Age and sex were not directly controlled between the AGRE and NIH groups. However, age- and sex-specific percentiles were derived from NHANES-III and were used for analysis of the lipid levels in the groups of individuals with ASD and typically developing individuals. Thus, the analyses did control for the variables of age and sex.

### Lipid testing

We measured lipid blood levels for the following number of AGRE subjects: 570 cholesterol (analyzed at NIH and Kennedy Krieger Inc. (KKI)), and 367 HDL, ApoA1, and ApoB (analyzed at NIH). Because the fasting status of the subjects were not recorded, triglyceride measurements and calculated LDL levels were not used. AGRE repository samples that had been divided into 25 µL aliquots and stored at −70 °C were transferred from the AGRE repository to the following two CLIA-certified laboratories: Department of Laboratory Medicine at the Clinical Center of the NIH (NIH) and the Biochemical Genetics Laboratory at the Hugo W. Moser Research Institute at KKI. The samples were stored at −70 °C at those sites and stored at −20 °C for ≤2 weeks before analysis. At KKI, cholesterol was quantified by dual column gas chromatography flame ionization detector + mass spectrometry (GC/MS) [48] (*n* = 190). The lowest and highest measurements of the GC/MS assay were cholesterol: 1 mg/dL–500 mg/dL. At NIH, cholesterol (*n* = 466), HDL (*n* = 367), ApoA1 (*n* = 367), and ApoB (*n* = 367) were quantified on a Vista analyzer. The lowest and highest measurements of the Vista analyzer assay were cholesterol: 50 mg/dL–600 mg/dL; HDL: 3 mg/dL–150 mg/dL; ApoA1: 19 mg/dL–600 mg/dL; ApoB: 26 mg/dL–400 mg/dL. No measurement exceeded the upper limits of the assays at KKI or NIH. The following numbers of subjects had levels below what could be measured by the analyzer at NIH (were “below assay”) and were assigned a value at the bottom assay’s range: 6 cholesterol values were assigned 50 mg/dL; 6 HDL were assigned 3 mg/dL; 3 ApoA1 were assigned 19 mg/dL; and 8 ApoB were assigned 26 mg/dL. No cholesterol measurement was below assay at KKI. Cholesterol was quantified at both NIH and KKI for 86 subjects and the NIH cholesterol results was used for all those subjects, with no results being <50 mg/dL. Therefore, of the 570 cholesterol levels that were used for analyses, 466 were analyzed on the Vista analyzer and 104 (18) were analyzed by GC/MS. For the 86 subjects who had cholesterol levels performed at both KKI and NIH, the NIH cholesterol mean and SD was 101.28 ± 41.0 mg/dL, while the KKI cholesterol mean was 136.51 ± 43.22 mg/dL, and the NIH-derived level values were used for most of the statistical analyses. No attempts were made to replicate the sterol and lipid testing results.

### Creation of lipid levels *Z*-scores (standard scores)

We created *Z*-scores using the lipid-level scores of the NHANES-III subjects to compare the lipid results of the AGRE subjects to a population without ASD diagnoses.

### Comparison of AGRE and NHANES-III subjects across age groups

We organized the AGRE and NHANES-III lipid-level data by age groups of 2–3-year intervals (4–5.9, 6–7.9, 8–10.9, 11–12.9, 13–14.9, 15–16.9, 17–18.9) to compare the ASD group to a USA survey population in order to determine whether there were patterns of lipid abnormalities that varied by age. Individuals <4.0 years and >18.9 were not included in the analyses.

### We assigned NHANES-III age- and sex-adjusted centile values for all the AGRE subjects’ lipid levels

We wanted to determine (1) whether the AGRE subjects have abnormal lipid levels, (2) lipid thresholds to separate the subjects into groups for analyses purposes, and (3) how the behavioral and physical characteristics of the individuals with abnormal lipid levels compare to the other AGRE subjects and control populations. Since mean lipid levels vary by age and sex in typically developing individuals [72, 73], we examined the data using age- and sex-adjusted centile values, rather than lipid level values or *Z*-scores. We assigned a centile value for each of the subjects’ cholesterol, HDL, ApoA1, and ApoB levels (and rounded the age and lipid value up or down as needed to reach the incremental lipid values on the tables). Subjects <4.0 years were assigned the age 4 year centile values. After observing the fold increases in the subjects’ lipid levels below the 5th centile (<5thCent), we divided the lipid data into two groups, those with centile values <5thCent and those with values ≥5th centile (≥5thCent) (<5thCent&≥5thCent-groups).

### Analysis to determine whether lipid patterns are stable across childhood and adolescence

We performed Pearson’s Chi-Square Test with Benjamini–Hochberg *p* value correction procedure (BHC) [81] to determine whether the proportions of subjects with <5thCent levels to those with ≥5thCent levels of cholesterol, HDL, ApoA1, and ApoB significantly differed across the 7 age intervals of 4–5.9, 6–7.9, 8–10.9, 11–12.9, 13–14.9, 15–16.9, and 17–18.9 years.

### Analyses of male to female sex ratios (M:F) within the lipid groups

We determined the M:F ratio for cholesterol (*n* = 570), HDL, ApoA1, and ApoB (all *n* = 367). We then performed Pearson’s Chi-Square Test with Yates’ Continuity Correction (YCC) analyses to determine whether the M:F were different between the <5thCent&≥5thCent-groups for each lipid (cholesterol, HDL, ApoA1, and ApoB).

### Measurement of 7DHC, lathosterol, desmosterol, and sitosterol and assessment for diagnoses of SLOS, lathosterolosis, desmosterolosis, and sitosterolemia

We tested serum samples from AGRE participants at the CLIA-certified laboratory at KKI. Post-squalene cholesterol precursors were identified and quantified by GC/MS [48]: 7DHC (*n* = 145), lathosterol (*n* = 190), and desmosterol (*n* = 145). Sitosterol, a plant sterol, was also quantified (*n* = 135). The chromatograms were capable of detecting evidence of 8DHC, but the amount could not be quantified. The lowest and highest measurements of the GC/MS assay were 7DHC, desmosterol, and lathosterol: all 0.01 µg/mL–500 µg/mL; sitosterol: 0.5 µg/mL–500 µg/mL (the units of measurements standard in the literature of the field of study were used for reporting; 10 µg/mL is equal to 1 mg/dL). No sample had a value below or above the assay range. We reviewed the sterol results and the small amount of available information regarding physical examination, growth parameters, and medications administered for clinical evidence of SLOS, lathosterolosis, desmosterolosis, and sitosterolemia.

### Examination of the relationship of lipid centiles to 7DHC, lathosterol, desmosterol, and sitosterol levels

We performed Kruskal–Wallis analyses with Benjamini–Hochberg correction procedure (Kruskal-Wallis-BHC) to determine whether individuals with <5thCent of cholesterol, HDL, ApoA1, and ApoB have lower amounts of 7DHC, lathosterol, desmosterol, and sitosterol than subjects with lipids ≥5thCent.

### Exploratory analyses of head circumference (OFC), weight, height, and body mass index (BMI)

We assigned OFC centiles (*n* = 149) using charts calculated by Roche [82], with subjects older than 18 years assigned centiles for individuals 18 years old and individuals with OFC values greater than the 95th centile were assigned a value of 95 and OFC less than the 5th centile were assigned a value of 5 for analysis purposes. We assigned weight (*n* = 91) and height (*n* = 84) centiles using values generated by the CDC [83], with subjects older than 20 years assigned centiles for individuals 20 years old and used the Medscape online calculators to calculate the centiles: https://reference.medscape.com/guide/medical-calculators. We calculated the BMI for the 65 subjects who had both weight and height measurements using the formula BMI = Weight (kg)/Height (m)^2^. We then performed Kruskal-Wallis-BHC of the four lipids with the four physical parameters.

### Kruskal-Wallis-BHC of cholesterol, HDL, ApoA1 and ApoB centiles with adaptive functioning and FSIQ

We performed Kruskal-Wallis-BHC of Vineland SS and FSIQ scores with cholesterol, HDL, ApoA1, and ApoB <5thCent&≥5thCent-groups.

### Kruskal-Wallis-BHC of HDL and ApoA1 centiles in the subset of subjects with 7DHC level quantification

We performed Kruskal-Wallis-BHC of Vineland SS with HDL and ApoA1 <5thCent&≥5thCent-groups in the subset of 37 subjects who had 7DHC level quantification to determine whether those with 7DHC levels would have findings similar to the analyses performed in all the subjects.

### Analyses of Vineland and FSIQ in the apolipoprotein groups

We performed Kruskal-Wallis-BHC with the subjects in each of the groups HABL, HAL, and HBL vs. all other subjects to see whether subjects in a group had lower Vineland or IQ scores than the rest of the subjects.

### Comparison of apolipoprotein patterns in cholesterol <5thCent AGRE subjects, NIH typical control subjects, and NHANES-III population

We compared the apolipoprotein patterns in 3 groups of subjects, 4–17.9 years, with cholesterol <5thCent: AGRE (*n* = 118), NIH typically developing control subjects (*n* = 24), and NHANES-III survey-weighted estimates of the percentage in the typical population (NHANES-III-SWEP) (*n* = 157) using the age- and sex-adjusted NHANES-III centiles. We performed Fisher’s Exact Test for Count Data Two-Sided to determine whether there was a significant difference in the distribution across the four apolipoprotein classifications (HABL, HAL, HBL, and no apolipoprotein disorder) between the AGRE and NIH groups. Statistical testing was not performed with the NHANES-III-SWEP group due to the weighted sampling scheme for the NHANES-III data.

### Statistical analyses

We used the statistical program R3.6.2 (2019–12–12). All significance tests were two-sided. We used Kruskal–Wallis tests to compare all continuous variables (sterol precursor level, functional outcomes, physical characteristics) across the lipid and apolipoprotein groups, due to the skewed nature of many of these variables. Where groups of variables were considered together, we used a BHC to account for multiple testing. Comparing a BHC *p* value to a significance level of 0.05 is equivalent to controlling the false discovery rate at 5%. When comparing binary variables across lipid and apolipoprotein groups, we used Pearson’s Chi-Square Test or Fisher’s Exact Test for Count Data Two-Sided when the number of individuals in a group was low. We calculated centiles and summary statistics from NHANES-III using survey-weighted methods to account for the design of the NHANES-III survey.

## Results

### Low cholesterol, HDL, ApoA1, and ApoB and elevated ApoA1 and ApoB in ASD

**Fig. 2:**
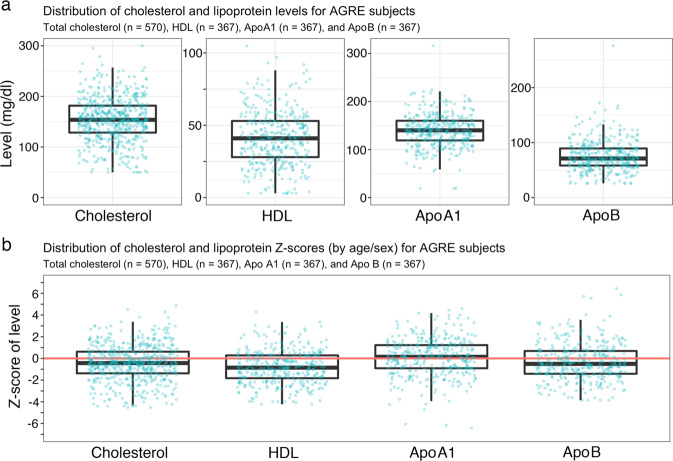
Distribution and *Z*-scores of cholesterol and lipoprotein levels in AGRE subjects. **a** Distributions of cholesterol (CHL) (*n* = 570), HDL, apolipoprotein A1 (ApoA1), and ApoB (each *n* = 367). Points represent levels (in mg/dL) for individual AGRE subjects. For the boxplots: centerline is median value, edges of box are upper and lower quartiles, whiskers extend to 1.5 times the interquartile range from the edge of the box. Points above and below the whiskers represent outliers. **b** Distributions of *Z*-scores for CHL (*n* = 570), HDL, ApoA1, and ApoB (each *n* = 367). *Z*-scores were calculated as *Z* = (level − mean)/(standard deviation), using age- and sex-specific means and standard deviations estimated using subjects from the National Health and Nutrition Examination Survey III (NHANES-III). Points represent *Z*-scores for individual AGRE subjects. For the boxplots: centerline is median value, edges of box are upper and lower quartiles, whiskers extend to 1.5 times the interquartile range from the edge of the box. Points above and below the whiskers represent outliers. Two outlying *Z*-scores are not shown on this plot: *Z* = 11.4 for ApoA1 and *Z* = 14.4 for ApoB.

**Fig. 3:**
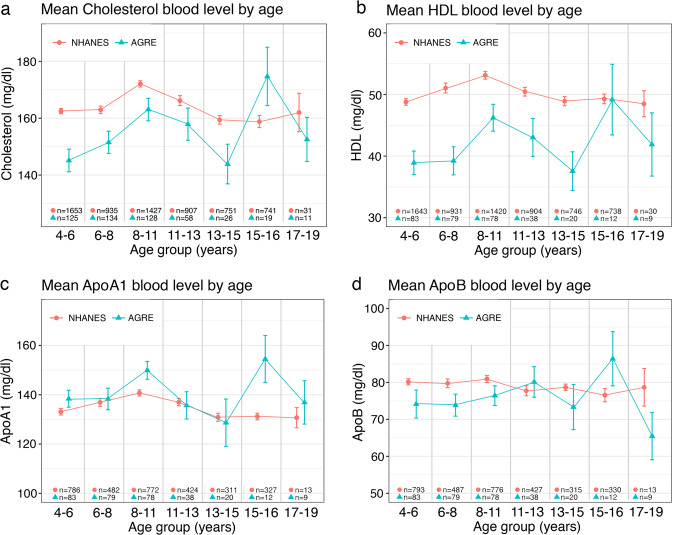
Distributions of NHANES-III and AGRE cholesterol and lipoprotein levels by age segments. **a** The distributions of cholesterol (CHL), HDL, apolipoprotein A1 (ApoA1), and ApoB by age segments, 4.0–18.9 years, between National Health and Nutrition Examination Survey III (NHANES-III) AGRE subjects: CHL 6445 and 500 AGRE subjects. **b** HDL 6412 NHANES-III and 312 AGRE subjects. **c** ApoA1 3115 NHANES-III and 312 AGRE subjects. **d** ApoB 3141 NHANES-III and 312 AGRE subjects. Center point is mean value, error bars extend one standard error (SE) of the mean in each direction. Mean and SE for NHANES-III group are calculated using survey-weighted methods.

We measured blood cholesterol, HDL, ApoA1, and ApoB levels of AGRE subjects: 570 for cholesterol and 367 for HDL, ApoA1, and ApoB (Fig. 2a) and then used the NHANES-III survey population data to create *Z*-scores using 2-year intervals (Fig. 2b). We also compared cholesterol, HDL, ApoA1, and ApoB levels between the AGRE and NHANES-III groups using 2–3-year intervals (Fig. 3a–d). The AGRE subjects had lower cholesterol and HDL levels than the NHANES-III subjects in all intervals from the ages of 4.0–18.9 years except 15.0–16.9 years, when all the AGRE lipid levels were elevated.

**Fig. 4:**
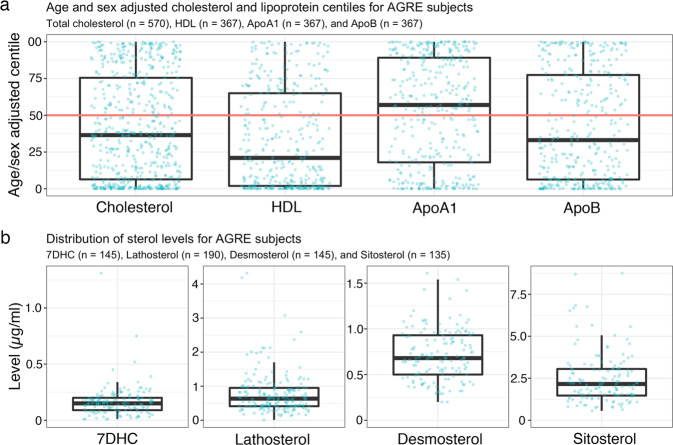
Distributions of AGRE cholesterol and lipoprotein centiles and sterol levels. **a** Distribution of cholesterol (*n* = 570), high-density lipoprotein cholesterol (HDL) (*n* = 367), apolipoprotein A1 (ApoA1) (*n* = 367), and apolipoprotein B (ApoB) (*n* = 367) centiles for AGRE subjects. **b** Distribution of 7-dehydrocholesterol (7DHC) (*n* = 145), lathosterol (*n* = 190), desmosterol (*n* = 145), and sitosterol (*n* = 135) blood levels. Points represent values for individual AGRE subjects. For the boxplots: centerline is median, edges of box are upper and lower quartiles, whiskers extend to 1.5 times the interquartile range from the edge of the box. Points above and below the whiskers represent outliers. Red line indicates 50th centile.

Using NHANES-III data, we created tables of age- and sex-specific lipid centiles in 2–3-year and 1-centile increments (Supplementary Tables 1–12). Because the subjects’ fasting status were not known, we did not use LDL and triglyceride data. We assigned NHANES-III centiles to the AGRE lipid levels to create age- and sex-adjusted centile values (Fig. 4a). We detected elevations in the number of individuals with levels <5thCent: cholesterol 23.0% (4.6-fold increase), HDL 31.6% (6.3-fold increase), ApoA1 16.9% (3.4-fold increase), and ApoB 22.1% (4.4-fold increase). There was a greater than 2-fold increase in individuals with apolipoprotein levels greater than the 95th centile (>95thCent), ApoA1 15% (3-fold increase), and ApoB 12.5% (2.5-fold increase) (Supplementary Table 13).

Using the centile values, we performed Pearson’s Chi-Square Test with BHC [81] and the proportions of AGRE subjects with <5thCent levels to ≥5thCent levels of cholesterol, HDL ApoA1, and ApoB did not significantly differ across 7 age intervals from age 4.0 to 18.9 years (all degree of freedom = 1: cholesterol *p* = 0.080, BHC = 0.322; HDL *p* = 0.654; BHC = 0.773; ApoA1 *p* = 0.773, BHC = 0.773; ApoB *p* = .280, BHC = 0.560).

### Hypocholesterolemic subjects with ASD had a lower M:F ratio

We performed Pearson’s Chi-Square Test with YCC analyses to determine whether the M:F ratio differed between the AGRE <5thCent&≥5thCent-groups of cholesterol (*n* = 570) and HDL, ApoA1, and ApoB (all *n* = 367) (Supplementary Table 13). The subjects with cholesterol <5thCent (*n* = 131) had significantly lower M:F ratio (2.9:1) than the subjects with cholesterol **≥**5thCent (*n* = 439) M:F ratio (5.8:1) (chi-square = 7.998, degree of freedom = 1; *p* = 0.005 after YCC). There were no significant differences in the M:F ratio with the HDL, ApoA1, and ApoB groups.

### No biochemical diagnosis of SLOS, lathosterolosis, desmosterolosis, or sitosterolemia

We measured 7DHC to determine whether any subject had a biochemical diagnosis of SLOS (Fig. 4b). Of the 145 AGRE subjects for whom 7DHC levels were measured, there were 13 subjects with 7DHC levels lower than the normal reference range of 0.04–0.36 µg/mL. All these participants had low lathosterol levels (0.01–1.32 µg/mL), indicating a low rate of sterol synthesis. Of the 145 subjects, 5 had levels of 7DHC >0.36 µg/mL requiring further evaluation to determine whether there were findings consistent with a clinical diagnosis of SLOS. Of the 5 subjects, 2 had mildly increased 7DHC levels (0.39 and 0.45 µg/mL) without detectable 8DHC and cholesterol levels of 163.0 mg/dL (*Z-*score + .21) and 300.0 mg/dL (*Z-*score +3.34), with the *Z-*score calculated using NHANES-III normally distributed cholesterol data. In addition, their increased 7DHC levels were consistent with their cholesterol centiles and, given their undetectable 8DHC, were unlikely to have SLOS. A third subject had a 7DHC level of 0.52 µg/mL, an unquantifiable trace amount of 8DHC, and a cholesterol level of 96 (*Z-*score −1.31). Physical examination, weight centile (95th), and head centile (95th) were inconsistent with the diagnosis of SLOS. At the time of medical history completion, 2 years after AGRE obtained the blood sample, that subject was treated with trazodone and buspirone medications, which are known to increase 7DHC levels in individuals without SLOS [84, 85]. The fourth subject had a 7DHC level of 0.75 µg/mL, a trace, unquantifiable level of 8DHC, and a cholesterol level 164.4 mg/dL (*Z-*score +0.25). The fifth subject had a 7DHC level of 1.31 µg/mL, an unquantifiable level of 8DHC, and a cholesterol level of 238.6 mg/dL (*Z-*score +1.94). While biochemically mild forms of SLOS cannot be excluded for the samples with increased 8DHC levels, other possibilities include carrier status for SLOS or medication effect, with the latter being the most likely in our subject population. All individuals with biochemically mild variants of SLOS diagnosed by the KKI laboratory have had one or more physical features of SLOS (RI Kelley, personal communication, January 22, 2021).

We also measured the levels of lathosterol (*n* = 190) (reference range 0.17–2.85 µg/mL), desmosterol (*n* = 145) (reference range 0.12–2.00 µg/mL), and sitosterol (*n* = 135) (reference range 0.03–6.17 µg/mL) to exclude other heritable disorders of post-squalene cholesterol synthesis (lathosterolosis, desmosterolosis) and impaired hepatic excretion of phytosterols (sitosterolemia) (Fig. 4b). Of the 5 subjects with lathosterol levels lower than the reference range, all had cholesterol centiles ≤2, indicating a low rate of sterol synthesis, a recognized, common cause of a low lathosterol level. Three subjects had modestly increased lathosterol levels (between 3.08 and 4.32 µg/mL), which could reflect the extreme of normal or a transient increase in the rate of cholesterol synthesis, e.g., recovery from infection. In addition, these mildly increased lathosterol levels are substantially less than the lowest lathosterol level found in a clinically mild case of lathosterolosis (32.0 µg/mL) [86]. No subject had a sitosterol level lower than the reference range and 6 had slightly elevated sitosterol levels (between 6.53 and 8.76 µg/mL), which might reflect the extreme of normal or an effect of diet or medication but which were inconsistent with a diagnosis of sitosterolemia.

### Decreased levels of 7DHC, lathosterol, desmosterol, and sitosterol in hypocholesterolemic ASD

We performed Kruskal-Wallis-BHC of 7DHC, lathosterol, desmosterol, and sitosterol levels between cholesterol, HDL, ApoA1, and ApoB <5thCent&≥5thCent-groups (Supplementary Table 14). Individuals with cholesterol <5thCent had significantly lower levels of 7DHC, lathosterol, and desmosterol (all *p* < 0.001; BHC *p* < 0.001) than subjects with lipids >5thCent. Subjects with ApoA1 <5thCent had significantly lower sitosterol (*p* = 0.016; BHC *p* = 0.044) and desmosterol levels (*p* = 0.023; BHC *p* = 0.052). Individuals with ApoB <5thCent had significantly lower 7DHC (*p* = 0.002; BHC *p* = 0.006), desmosterol levels (*p* **<** 0.001; BHC *p* **<** 0.001), and lathosterol levels (*p* = 0.026; BHC *p* = 0.052) (Supplementary Table 15).

### Trend toward lower BMI with low ApoB

We performed exploratory Kruskal-Wallis-BHC of OFC, height and weight centiles and BMI between cholesterol, HDL, ApoA1, and ApoB <5thCent&≥5thCent-groups. There were no significant results, although there was a trend toward significance of ApoB **<**5thCent (*n* = 12) having a lower BMI (*p* = 0.006; BHC *p* = 0.095) (Supplementary Table 16).

### Lower life skills but no difference in IQ in subjects with low HDL or ApoA1 levels

**Fig. 5:**
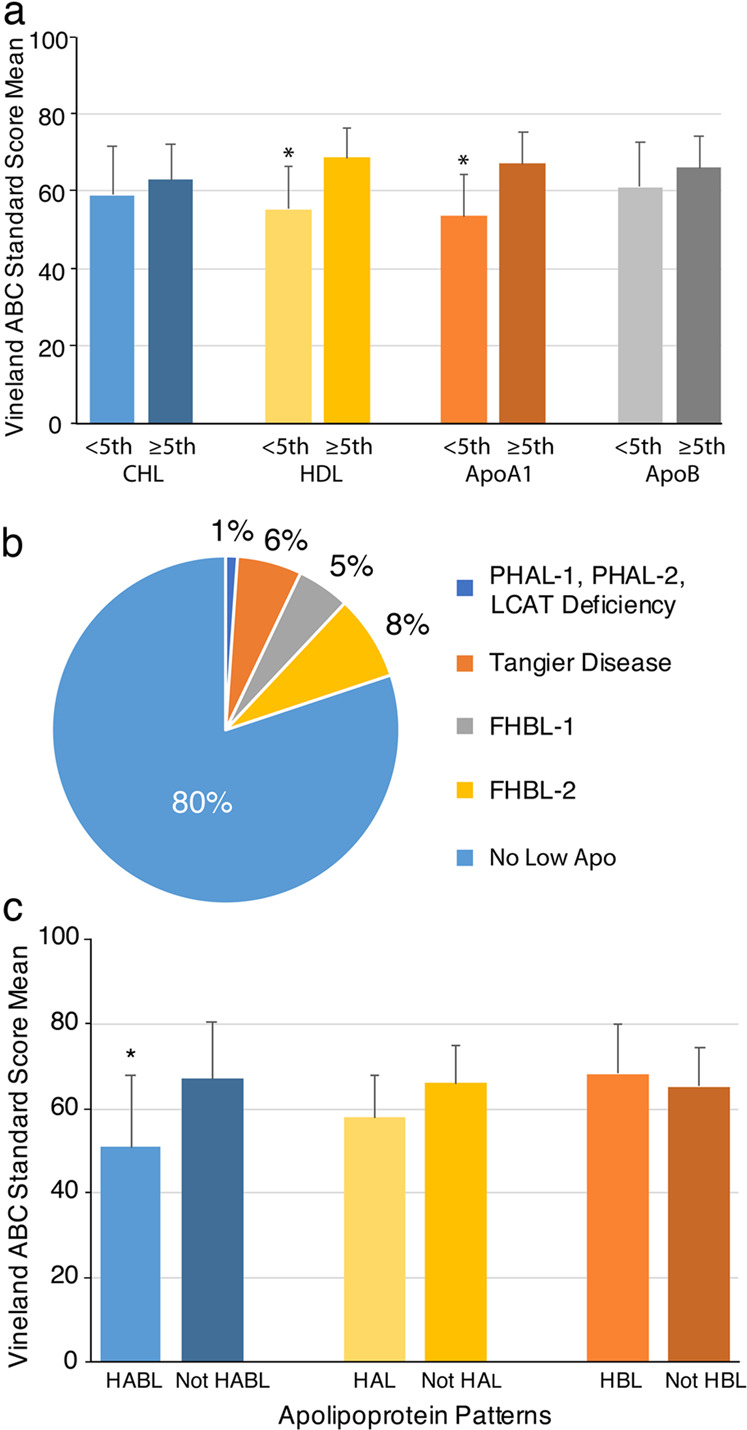
AGRE subjects with low lipoprotein levels have lower adaptive functioning and patterns approximating hypoapolipoprotein disorders. **a** Kruskal–Wallis analyses with Benjamini–Hochberg procedure (KW-BHC) were performed with Vineland ABC standard scores (V-ABC-SS) between groups with cholesterol (CHL) (*n* = 404), HDL, apolipoprotein A1 (ApoA1), and ApoB (each *n* = 268) levels < 5th centile (<5thCent) & ≥ 5th centile (≥5thCent). Error bars extend 1 standard deviation (SD) above the mean. *V-ABC-SS were significantly lower (had more impaired life skills) in individuals with HDL < 5thCent (*p* < 0.001; BHC *p* < 0.001) and ApoA1 < 5thCent (*p* = 0.001; BHC *p* = 0.003). **b** Lipids were categorized into disorder approximations: primary hypoalphalipoproteinemia-1 (PHAL-1), primary hypoalphalipoproteinemia-2 (PHAL-2) and lecithin:CHL acyltransferase (LCAT) deficiency (low ApoA1, low HDL, normal CHL, normal ApoB); Tangier disease (low ApoA1, low HDL, low CHL, normal ApoB); familial hypobetalipoproteinemia-1 (FHBL-1) (low ApoB, low CHL, normal HDL, normal ApoA1); combined hypolipidemia (also known as familial hypobetalipoproteinemia-2, FHBL-2) (low ApoA1, low ApoB, low HDL, low CHL). **c** KW-BHC of V-ABC-SS between groups vs. all others: HABL (ApoA1 < 5thCent & ApoB < 5thCent, *n* = 22); HAL (ApoA1 < 5thCent & ApoB ≥ 5thCent, *n* = 15); HBL (ApoA1 ≥ 5thCent & ApoB < 5thCent, *n* = 34). Error bars extend 1 SD above the mean. *Individuals with HABL had lower V-ABC-SS (*p* = 0.003; BHC *p* = 0.017).

We performed Kruskal-Wallis-BHC of Vineland-ABC SS [69] and the FSIQ between cholesterol, HDL, ApoA1, and ApoB <5thCent&≥5thCent-groups. The Vineland-ABC SS were significantly lower (indicating more impairment in life skills) in individuals with levels <5thCent for HDL (*p* < 0.001; BHC *p* < 0.001) and ApoA1 (*p* = 0.001; BHC *p* = 0.003) while the analyses of cholesterol and ApoB were not significant (Fig. 5a). Since the HDL and ApoA1 analyses with Vineland-ABC were significant, we performed Kruskal-Wallis-BHC of the 4 Vineland domain SS with the HDL and ApoA1 <5thCent&≥5thCent-groups. The HDL <5thCent group had significantly lower SS on Vineland Communication (*p* = 0.002; BHC *p* = 0.003), Daily Living Skills (*p* < 0.001; BHC *p* = 0.001), and Socialization (*p* < 0.001; BHC *p* < 0.001). The ApoA1 <5thCent group had significantly lower SS on Daily Living Skills (*p* = 0.002; BHC *p* = 0.003) and Socialization (*p* < 0.001; BHC *p* = 0.001). Kruskal-Wallis-BHC of FSIQ SS with cholesterol, HDL, ApoA1, and ApoB <5thCent&≥5thCent-groups were not significant (Supplementary Table 17). Because we analyzed 86 subjects’ cholesterol levels at both NIH and KKI and knew that the NIH cholesterol mean was lower (101.28 ± 41.0 mg/dL) than the KKI cholesterol mean (136.51 ± 43.22 mg/dL), we performed Kruskal-Wallis-BHC of Vineland-ABC SS and the FSIQ between the 466 cholesterol <5thCent&≥5thCent-groups after excluding the 104 subjects who had cholesterol performed only at KKI, and the uncorrected *p* values were also not significant (Vineland-ABC *p* = 0.078, FSIQ SS *p* = 1.00). All HDL, ApoA1, and APOB measurements were made at NIH.

Because 7DHC was not measured in all samples, to determine whether undiagnosed elevated 7DHC levels consistent with SLOS might be responsible for the significant results of the Vineland with HDL and ApoA1, we performed the same analyses of Vineland-ABC with the HDL and ApoA1 <5thCent&≥5thCent-groups in the subset of 37 subjects who underwent 7DHC analyses and did not have SLOS. The differences of the Vineland-ABC means between all 286 subjects and the 37 7DHC-tested subjects varied between 1.9 and 6.8 mg/dL, and there was a trend toward significant differences between the Vineland-ABC SS of the HDL (*p* = 0.082; BHC *p* = 0.082) and ApoA1 <5thCent&≥5thCent-groups (*p* = 0.080; BHC *p* = 0.082) (Supplementary Table 18).

### Low apolipoproteins suggestive of clinical disorders in ASD

Due to the lack of triglyceride and LDL measurements, we could not determine how many subjects had clinical lipid disorders. However, 19.9% of the AGRE subjects had patterns similar to the following hypolipidemic clinical conditions (Fig. 5b): primary hypoalphalipoproteinemia-1 (Online Mendelian Inheritance of Man [OMIM®] #604091, https://omim.org/), primary hypoalphalipoproteinemia-2 (OMIM #618463), and lecithin:cholesterol acyltransferase deficiency (OMIM #245900) (low ApoA1, low HDL, normal cholesterol, normal ApoB): 4 (4 M, 0 F) (1.1% of subjects); Tangier disease (OMIM #205400) (low ApoA1, low HDL, low cholesterol, normal ApoB): 22 (17 M, 5 F) (6.0%); familial hypobetalipoproteinemia-1 (OMIM #615558) (low ApoB, low cholesterol, normal HDL, normal ApoA1): 18 (12 M, 6 F) (4.9%); and combined hypolipidemia (also known as familial hypobetalipoproteinemia-2, OMIM #605019) (low ApoA1, low ApoB, low HDL, low cholesterol): 29 (24 M, 5 F) (7.9%).

Of the 367 AGRE subjects, 17% had ApoA1 <5thCent and 22% had ApoB **<**5thCent, and we organized the subjects into apolipoprotein groups: combined hypolipidemia (HABL) (ApoA1 <5thCent, ApoB <5thCent): 34 (28 M, 6 F) (9% of subjects); hypoalphalipoproteinemia (HAL) (ApoA1 <5thCent, ApoB ≥5thCent): 28 (23 M, 5 F) (8%); hypobetalipoproteinemia (HBL) (ApoA1 ≥5thCent, ApoB <5thCent): 47 (35 M, 12 F) (13%); Normal (ApoA1 ≥5thCent, ApoB ≥5thCent): 258 (219 M, 39 F) (70%) (Supplementary Table 19).

### Lower life skills in ASD with low ApoA1 plus low ApoB

We performed Kruskal-Wallis–BHC of the Vineland-ABC SS in each group, HABL (*n* = 22), HAL (*n* = 15), and HBL (*n* = 34) vs. all other AGRE subjects (Supplementary Table 20). The individuals in the HABL group had significantly lower Vineland-ABC SS (indicating lower adaptive functioning) than individuals not in the HABL group (*p* = 0.003; BHC *p* = 0.017) (Fig. 5c). Since the analysis of the HABL group was significant, we performed Kruskal-Wallis-BHC of the four Vineland Domain SS with the HABL group vs. all other subjects. The HABL group had lower scores in Communication (*p* = 0.033; BHC *p* = 0.044), Daily Living Skills (*p* = 0.002; BHC *p* = 0.003), and Socialization (*p* = 0.002; BHC *p* = 0.003). There were no significant differences in Vineland-ABC SS between either the HAL group or the HBL group and the remainder of the subjects.

### Different apolipoprotein patterns in ASD and typical subjects

We compared ApoA1 and ApoB levels in the 3 groups, age 4–17.9 years, with cholesterol <5thCent based upon age- and sex-adjusted NHANES-III centiles: AGRE (*n* = 118), NIH typically developing control subjects (*n* = 24), and NHANES-III survey-weighted estimates of the percentage in the typical population (NHANES-III-SWEP) (*n* = 157) (Table 1). The hypocholesterolemic AGRE subjects had the highest percentage with ApoA1 <5thCent (AGRE 45%, NIH 17%, NHANES-III-SWEP 17%), while the hypocholesterolemic NIH control group had the highest percentage with ApoB <5thCent (AGRE 51%, NIH 71%, NHANES-III-SWEP 45%). We then grouped all subjects into the categories of HABL, HAL, HBL, and Normal. The ASD subjects had the highest percentage of HABL (AGRE 26%, NIH 4%, NHANES-III-SWEP 6%) and HAL (AGRE 19%, NIH 12%, NHANES-III-SWEP 11%), while the typically developing children had the highest percentage of HBL (AGRE 25%, NIH 67%, NHANES-III-SWEP 38%). We performed 2-sided Fisher’s Exact Test for Count Data Two-Sided and there was a significant difference in the distribution across the 4 apolipoprotein classifications between the hypocholesterolemic AGRE and NIH groups (*p* = 0.0008). We did not perform statistical testing with the NHANES-III-SWEP group due to the weighted sampling scheme for the NHANES-III data.

**Table 1:** Apolipoprotein patterns in AGRE, typically developing, and NHANES-III subjects’ age 4.0–17.9 years with cholesterol levels less than the 5th centile.

118 independent AGRE children with ASD cholesterol levels <5thCent^a^	
53 (45%) cholesterol <5thCent subjects had ApoA1 levels <5thCent	
60 (51%) cholesterol <5thCent subjects had ApoB levels <5thCent	
31 of 118 (26%) AGRE	had both ApoA1 and ApoB levels <5thCent (HABL)
22 of 118 (19%) AGRE	had ApoA1 levels <5thCent and ApoB ≥5thCent (HAL)
29 of 118 (25%) AGRE	had ApoB levels <5thCent and ApoA1 ≥5thCent (HBL)
36 of 118 (31%) AGRE	had both ApoA1 and ApoB levels ≥5thCent (Normal)
24 NIH typically developing children with cholesterol <5thCent^a^	
4 (17%) cholesterol <5thCent subjects had ApoA1 levels <5thCent	
17 (71%) cholesterol <5thCent subjects had ApoB levels <5thCent	
1 of 24 (4%) NIH	had both ApoA1 and ApoB levels <5thCent (HABL)
3 of 24 (13%) NIH	had ApoA1 levels <5thCent and ApoB ≥5thCent (HAL)
16 of 24 (67%) NIH	had ApoB levels <5thCent and ApoA1 ≥5thCent (HBL)
4 of 24 (17%) NIH	had both ApoA1 and ApoB levels ≥5thCent (Normal)
157 NHANES III children with cholesterol levels <SWEP 5^th^Cent	
27 (17%) cholesterol <SWEP 5thCent subjects had ApoA1 levels <5thCent	
70 (45%) cholesterol <SWEP 5thCent subjects had ApoB levels <5thCent	
10 of 157 (6%) SWEP	had both ApoA1 and ApoB levels <5thCent (HABL)
17 of 157 (11%) SWEP	had ApoA1 levels <5thCent and ApoB ≥5thCent (HAL)
60 of 157 (38%) SWEP	had ApoB levels <5thCent and ApoA1 ≥5thCent (HBL)
70 of 157 (45%) SWEP	had both ApoA1 and ApoB levels ≥5thCent (Normal)

*AGRE* Autism Genetic Research Exchange, *Apo* apolipoprotein, *ApoA1* apolipoprotein A1, *ApoB* apolipoprotein B, *ASD* autism spectrum disorder, *Cent* centile, *HABL* had both ApoA1 and ApoB levels <5thCent, *HAL* had ApoA1 levels <5thCent and ApoB ≥5thCent, *HBL* had ApoB levels <5thCent and ApoA1 ≥5thCent, *NHANES III* National Health and Nutrition Examination Survey III, *NIH* National Institutes of Health, *SWEP* survey-weighted estimate of the percentage in the typically developing population.

^a^There was a significant difference in the distribution across the 4 apolipoprotein classifications between the AGRE and NIH groups (2-sided Fisher’s Test *p* = 0.0008). Statistical testing was not performed with the NHANES-III group due to the weighted sampling scheme for the NHANES-III data.

## Discussion

We identified a 3.4–6.3-fold increase in AGRE subjects with cholesterol, HDL, ApoA1, and ApoB levels **<**5thCent and a 2.5–3-fold increase in subjects with ApoA1 and ApoB levels >95thCent. Despite the increased percentage with hypocholesterolemia, no subject had a finding consistent with SLOS or other disorders of post-squalene cholesterol synthesis, confirming that SLOS is rare within ASD in the absence of cardinal clinical features. Because subjects were selected only from multiplex families, we would expect a greater representation of autosomal-recessive genetic disorders. The fact that no SLOS was identified indicates that unrecognized SLOS probably represents <0.7% of ASD and more likely <0.2%. This is consonant with the epidemiology of SLOS and the distribution of *DHCR7* variants. Subjects with abnormally low cholesterol levels had statistically lower levels of 7DHC, lathosterol, and desmosterol (all with BHC <0.001). This pattern indicates that low cholesterol levels are not due to gastrointestinal loss or diet restrictions since these conditions will have increased levels of cholesterol precursors, especially lathosterol [87, 88]. Instead, the data suggest that subjects with ASD who have low cholesterol levels have intrinsically reduced cholesterol synthesis.

The significantly lower M:F ratio of 2.9:1 in the cholesterol <5thCent group suggests that there might be a genetic etiology that affects individuals with very low cholesterol levels that could be present during the prenatal period. There were 64% more subjects with low cholesterol values than with HDL or apolipoprotein measurements, so the significance might be due to the larger number of cholesterol measurements. The proportions of AGRE subjects with <5thCent levels to those with ≥5thCent levels for cholesterol, HDL ApoA1, and ApoB did not significantly differ across the 7 age intervals, which suggests that the onset of the lipid abnormalities occurs prior to the age of 4.0 years. Although we observed a peak in the 4 lipid levels during age 15.0–16.9 years that could indicate ASD-related lipid dysregulation related to puberty, the data may have been skewed due to the small number of subjects in that interval. Based on the natural history of individuals with SLOS, we hypothesized that individuals with low lipid levels would have growth delay and decreased OFC. However, we detected only a trend toward significance with ApoB and BMI, which is consistent with familial hypobetalipoproteinemia presenting with low BMI [89]. Furthermore, the HABL group (*n* = 17), with a mean cholesterol level of 80 mg/dL, had a mean OFC at the 83rd centile.

Subjects with HDL <5thCent had significantly lower overall adaptive abilities, as well as lower skills in the areas of communication, daily living, and socialization than subjects with HDL **≥**5thCent levels, just as individuals with ApoA1 <5thCent had significantly lower overall adaptive abilities, skills of daily living, and socialization than the ApoA1 ≥5thCent subjects (all with BHC ≤0.003). In the subjects who had 7DHC measured (and shown to not have SLOS), the differences between the Vineland-ABC means of all subjects and the 7DHC-tested subjects were <7 mg/dL. The Kruskal-Wallis–BHC of Vineland-ABC with the HDL and ApoA1 <5thCent&≥5thCent-groups trended toward but did not reach statistical significance, which was likely secondary to the smaller number of subjects (all subjects *n* = 286, 7DHC tested *n* = 37). Therefore, it is unlikely that 7DHC is related to decreased HDL and ApoA1 levels or the associated adaptive functioning deficits. ApoA1 is the primary protein constituent of HDL. Therefore, our findings could be due to a primary biological mechanism that affects either ApoA1 and/or HDL production, regulation, or function. The brain is dependent upon ApoA1 being transported from the periphery to the CNS, so a deficit of ApoA1 in the periphery suggests that a deficit could exist in the CNS as well. The CNS relies on its own production of cholesterol and HDL-like particles, so deficits of cholesterol and HDL in the periphery might not be as likely to also exist in the CNS. In addition, the complex regulation of lipid gene expression in the periphery could differ from regulation of the same genes in the CNS.

The AGRE subjects with cholesterol <5thCent had a mean cholesterol of 96 mg/dL and the analysis of the differences in the Vineland-ABC scores between the cholesterol <5thCent&≥5thCent-groups was not significant. However, in analyses of the subset of subjects who had apolipoprotein testing and Vineland scores, the HABL subjects demonstrated more impairment in adaptive functioning than the rest of the subjects and this might have been related to their lower cholesterol levels and centiles (mean of 78 mg/dL, plus 3 cholesterol measurements below what the assay could measure and assigned the value 50 mg/dL, mean centile 2.6), while the HAL and HBL groups, which did not have adaptive function impairment, had mean cholesterol levels of 113 and 118 mg/dL, respectively, and mean centiles 9.4 and 12.4, respectively. The HABL group also had lower ApoA1 levels and centiles (mean of 83 mg/dL with 3 measurements assigned the value of 19 mg/dL, mean centile 1.1) than the HAL group (mean of 97 mg/dL with no measures below assay, mean centile 1.9), so there may be a threshold effect with ApoA1 levels that leads to ASD symptoms since ApoA1 has multiple roles within the CNS and has been implicated in numerous neurologic disorders. For example, *APOA1* gene variants could decrease ApoA1 levels in the CNS and present with low ApoA1 as a biomarker in blood. In addition to HABL being the only of the three apolipoprotein classifications that exhibited significant differences in Vineland SS, further supporting the potential biological importance of HABL is our finding that there was a significant difference in the distribution across the 4 apolipoprotein categories between the hypocholesterolemic AGRE (*n* = 118) and hypocholesterolemic typically developing subjects (*n* = 24) (*p* = 0.0008).

The ApoB <5thCent findings in the typical subjects, and in at least some of the AGRE subjects, are most likely due to familial hypobetalipoproteinemia, with a prevalence of 1:1000–1:3000 in the general population. The AGRE subjects with HABL might have familial combined hypolipidemia, which is primarily secondary to loss-of-function mutations in the genes of the angiopoietin-like proteins, including *ANGPTL3* [90–92], which codes for a glycoprotein with roles in angiogenesis, tissue remodeling, inflammation, and inhibition of lipoprotein lipase (LPL) catalytic activity [93–95]. LPL hydrolyzes circulating chylomicrons and very-low-density lipoprotein, is a regulator of brain energy balance, is strongly expressed in the hypothalamus, hippocampus, and striatum, and *LPL* variants are associated with neurite pathology and Alzheimer disease [96–98]. *LPL* was identified as a gene affected in multiple hyperlipidemic conditions including familial combined hyperlipidemia [99] and is an autism candidate gene [100]. Increased plasma LPL activity has been detected in ASD [61] and an analysis of dyslipidemia in ASD identified sex-differentially expressed, neurodevelopmentally co-regulated, ASD segregating deleterious variants of *LPL* [65].

Since 90% of miRNA are carried by HDL and reduced blood HDL levels have been shown to reduce the number of particles available for the transport of miRNA [5], the lower adaptive impairment detected in the HDL **<**5thCent group might be related to decreased amounts of circulating miRNA. HDL and LDL also transport cholesterol to the adrenal gland for the production of neuroactive steroids, which are then transported to the CNS, so the lower HDL levels could also theoretically lower adaptive functioning due to neuroactive steroid deficits or from deficits of other things that the lipoproteins transport to the brain. However, the adaptive skill deficits in the HABL group do not appear related to low blood HDL levels, since the HABL group had a higher mean HDL centile than the HAL group and the HAL group did not have adaptive functioning deficits.

Findings of lipid abnormalities have been inconsistent in reported ASD studies [56–59, 64], although this might be partially due to varying dietary intake in countries where the studies were performed. However, our detection of a 2.5–3-fold increase in individuals with elevated apolipoprotein levels is consistent with a recent study using USA healthcare insurance claims that identified dyslipidemia diagnoses in 6.6% of individuals with ASD treated for primarily hyperlipidemic conditions [65]. A potential cause of both hypolipidemic and hyperlipidemic states in ASD is that variants in one gene can result in the gene’s gain of function or loss of function. For example, PCSK9 is important in regulation of lipoproteins and neuronal apoptosis [101] and has abnormal function in individuals with fragile X syndrome [40, 97]. Gain-of-function gene variants of *PCSK9* occur with familial combined hypercholesterolemia, in which individuals have increases in plasma total cholesterol, ApoB, and/or triglyceride levels, while loss-of-function variants occur with familial hypobetalipoproteinemia-1. Individuals with familial combined hyperlipidemia-1 have elevated levels of PCSK9 [102], lathosterol, and desmosterol [103], so individuals with low ApoB levels might have low lathosterol and desmosterol levels, as we detected in our subjects.

While prior studies have shown abnormal lipid levels in individuals with ASD, we present new evidence for a disorder of cholesterol homeostasis in ASD other than SLOS. We demonstrated that individuals with ASD from multiplex families have 3.4–4.4-fold increase in the number of individuals with abnormally low levels of ApoA1 and ApoB, respectively, and 19.9% of the subjects had cholesterol, HDL, and apolipoprotein patterns that was similar to hypolipidemic clinical disorders. We also demonstrated that hypocholesterolemic AGRE subjects had a 2.6-fold increase in individuals with ApoA1 <5thCent, a >4-fold increase in individuals with both ApoA1 <5thCent plus ApoB <5thCent and an apolipoprotein pattern significantly different from typically developing individuals. Furthermore, we demonstrated that individuals with ASD with either HDL <5thCent or ApoA1 <5thCent or both ApoA1 <5thCent plus ApoB <5thCent have lower adaptive functioning, which points to various potential biological mechanisms that might, in one individual, cause CNS connectivity abnormalities, disrupt miRNA and neuroactive steroid transport, and lead to the diagnosis of ASD and adaptive skill deficits, which requires future study.

### Limitations of the study

Limitations include that (1) 7DHC was not quantified in all samples; (2) we lacked information regarding parental lipoprotein profiles, which could help identify inherited lipoprotein conditions; (3) the subjects were not fasting at the time of the blood draws so we could not perform triglyceride analyses or calculate the LDL levels using a triglyceride-dependent formula; (4) we had limited clinical information about diet, medications, and supplements that were being taken at the time of the blood draw, as well as other conditions, such as chronic diarrhea that could underlie the abnormal lipid pattern detected in our subjects or contribute to elevated 7DHC plus 8DHC levels; (5) we had limited physical parameter information to be able to determine whether there are unique physical characteristics to individuals with lipid levels <5thCent and to be able to determine whether two subjects who had both elevated 7DHC and 8DHC levels had physical features of SLOS; (6) analyses were performed only on blood samples, while lipid metabolism and regulation are different in the periphery than in the CNS; (7) the blood samples were often obtained from the children at different ages than the physical exams, medical interviews, and behavioral forms; and (8) the samples were from multiplex families so we cannot extrapolate as to the degree of sterol and lipid abnormalities in subjects from simplex ASD families.

## Supplementary information

Supplementary Tables 13 to 20_8–12–2021

Index of Supplementary Tables 1 to 20_8–12–2021

Supp_Table_01_NHANES-III_percentiles_cholesterol_4.0–16.9 yrs_Tierney_Transl_Psychiatry_2021

Supp_Table_02_NHANES-III_percentiles_HDL_4.0–16.9 yrs_Tierney_Transl_Psychiatry_2021

Supp_Table_03_NHANES-III_percentiles_ApoA1_4.0–16.9 yrs_Tierney_Transl_Psychiatry_2021

Supp_Table_04_NHANES-III_percentiles_ApoB_4.0–16.9 yrs_Tierney_Transl_Psychiatry_2021

Supp_Table_05_NHANES-III_percentiles_calculated_LDL_4.0–16.9 yrs_Tierney_Transl_Psychiatry_2021

Supp_Table_06_NHANES-III_percentiles_triglycerides_4.0–16.9 yrs_Tierney_Transl_Psychiatry_2021

Supp_Table_07_NHANES-III_percentiles_cholesterol_17.0–90.0 yrs_Tierney_Transl_Psychiatry_2021

Supp_Table_08_NHANES-III_percentiles_HDL_17.0–90_yrs_Tierney_Transl_Psychiatry_2021

Supp_Table_09_NHANES-III_percentiles_ApoA1_17.0–90.0 yrs_Tierney_Transl_Psychiatry_2021

Supp_Table_10_NHANES-III_percentiles_ApoB_17.0 years-90.0 yrs_Tierney_Transl_Psychiatry_2021

Supp_Table_11_NHANES-III_percentiles_calculated_LDL_17.0–90.0 yrs_Tierney_Transl_Psychiatry_2021

Supp_Table_12_NHANES-III_percentiles_triglycerides_17.0–90.0 yrs_Tierney_Transl_Psychiatry_2021

## Data Availability

The results of the lipid and sterol testing will be transmitted to the Autism Genetic Resource Exchange (AGRE) and AGRE can make the data available to approved researchers in addition to their behavioral data that is already available. AGRE has a limited supply of serum and plasma from the AGRE subjects who participated in this study. The tables of the age-and-sex-specific centiles for cholesterol, HDL, ApoA1 and ApoB LDL, and triglyceride plasma levels for individuals aged ≥4 years that were generated from NHANES-III are published as Supplementary Tables 1–12 to this article. NHANES-III data files are available for download at www.cdc.gov. NIH data and biosamples may be available upon request.
