# Supplementary material for: Sterol and lipid analyses identifies hypolipidemia and apolipoprotein disorders in autism associated with adaptive functioning deficits
Source: Transl Psychiatry. 2021 Sep 9;11:471. doi: 10.1038/s41398-021-01580-8 (PMC8429516; doi:10.1038/s41398-021-01580-8)
Supplement: Supplementary file 1 — Supplementary Tables 13 to 20_8–12–2021 [file 41398_2021_1580_MOESM1_ESM.docx]

# Supplementary Table 13: AGRE subjects with low and high levels of cholesterol, HDL, ApoA1, and ApoB.

| Lipid (grouping variable) | Centile groups for lipids | Number of subjects Male, Female | Sex ratio | Percentage of subjects with lipids in the category | Fold increase in lipid centile |
| --- | --- | --- | --- | --- | --- |
| Cholesterol (n = 570) | <5^th^Cent | (n = 131)  M 97, F 34 | 2.9:1* | 23.0% | 4.6 |
|  | ≥5^th^ Cent | (n = 439)  M 374, F 65 | 5.8:1* | 77% |  |
|  | >95^th^ Cent | (n = 49)  M 41, F 8 | 5.1:1 | 8.6% | 1.7 |
| HDL  (n = 367) | <5^th^ Cent | (n = 116)  M 95, F 21 | 4.5:1 | 31.6% | 6.3 |
|  | ≥5^th^ Cent | (n=251)  M 210, F 41 | 5.1:1 | 68.4% |  |
|  | >95^th^ Cent | (n = 20)  M 18, F 2 | 9:1 | 5.4% | 1.1 |
| ApoA1 (n = 367) | <5^th^ Cent | (n = 62)  M 51, F 11 | 4.6:1 | 16.9% | 3.4 |
|  | ≥5^th^ Cent | (n = 305)  M 254, F 51 | 5.0:1 | 83.1% |  |
|  | >95^th^ Cent | (n = 55)  M 49, F 6 | 8.2:1 | 15.0% | 3.0 |
| ApoB (n = 367) | <5^th^ Cent | (n = 81)  M 63, F 18 | 3.5:1 | 22.1% | 4.4 |
|  | ≥5^th^ Cent | (n=286)  M 242, F 44 | 5.5:1 | 77.9% |  |
|  | >95^th^ Cent | (n = 46)  M 38, F 8 | 4.8:1 | 12.5% | 2.5 |

* Sex ratio was significantly different between the <5thCent and >5thCent groups by Pearson’s Chi-Square Test with Yates’ Continuity Correction (YCC) (chi-square=7.680, degree of freedom=1; p=.006 after YCC).

a AGRE, Autism Genetic Research Exchange; ApoA1, apolipoprotein A1; ApoB, apolipoprotein B; Cent, centile; HDL, high-density lipoprotein cholesterol; M, male; F, female; %, percent.

# Supplementary Table 14: AGRE subject cholesterol and lipoprotein characterization: subject number, <5th and >5^th^ centile groups’ mean levels, age, assay information.

| Lipid | All subjects’ levels Mean + SD (mg/dL) Male, Female | All subjects’ centile Mean + SD | <5thCent group | | | >5thCent group | | | % with centile zero | Assay floor value  (mg/dL) † [below assay] |
| --- | --- | --- | --- | --- | --- | --- | --- | --- | --- | --- |
|  |  |  | Level Mean + SD  (mg/dL) | Centile Mean + SD | Age Mean + SD | Level Mean + SD  (mg/dL) | Centile Mean + SD | Age Mean + SD |  |  |
| CHL | 153.67 + 43.86  (n = 570)  M 471, F 99 | 41.69 + 34.93 | 94.98 + 22.19  (n = 131)  M 97, F 34 | 0.98 + 1.19 | 8.1 + 4.5 | 171.18 + 31.87  (n = 439)  M 374, F 65 | 53.83 + 30.68 | 8.9 + 4.6 | 10% | 50 mg/dL  [6] |
| HDL | 41.27 + 18.60  (n = 367)  M 305, F 62 | 33.22 + 33.45 | 20.82 + 8.36  (n = 116)  M 95, F 21 | 1.06 + 1.33 | 8.7 + 5.5 | 50.72 + 13.81  (n = 251)  M 210, F 41 | 48.08 + 30.59 | 9.2 + 4.6 | 10% | 3 mg/dL [6] |
| ApoA1 | 139.90 + 34.67  (n = 367)  M 305, F 62 | 53.41 + 35.95 | 89.55 + 20.77  (n = 62)  M 51, F 11 | 1.46 + 1.59 | 8.5 + 4.8 | 150.14 + 27.17  (n = 305)  M 254, F 51 | 63.96 + 29.89 | 9.1 + 5.0 | 6% | 19 mg/dL [3] |
| ApoB | 75.73 + 28.35  (n = 367)  M 305, F 62 | 41.70 + 36.31 | 43.97 + 9.92  (n = 81)  M 63, F 18 | 1.25 + 1.50 | 7.9 + 4.0 | 84.73 + 25.23  (n = 286)  M 242, F 44 | 53.16 + 33.11 | 9.3 + 5.1 | 9% | 26 mg/dL [10] |

† [ ] Indicates number of subjects with levels below measurable range of the lipid assay.

a AGRE, Autism Genetic Research Exchange; ApoA1, apolipoprotein A1; ApoB, apolipoprotein B; Cent, centile; CHL, cholesterol; HDL, high-density lipoprotein cholesterol; NIH, National Institutes of Health; SD, standard deviation; %, percentage.

# Supplementary Table 15: Kruskal Wallis analyses of <5^th^ and >5^th^ centile groups of cholesterol, HDL, ApoA1 and ApoB with 7DHC, lathosterol, desmosterol and sitosterol in AGRE subjects.

| Lipid (Grouping variable) | Sterol | | Sterol Mean + SD  (Lipid <5thCent group) (µg/mL) | Sterol Mean + SD  (Lipid >5thCent group) (µg/mL) | Kruskal Wallis p value | Significance p value after BHC† |
| --- | --- | --- | --- | --- | --- | --- |
| CHL | 7DHC | n = 145 | 0.117 + 0.093  (n = 55) | 0.195 + 0.153  (n = 90) | <.001 | <.001* |
|  | Lathosterol | n = 190 | 0.594 + 0.647  (n = 81) | 0.918 + 0.546  (n = 109) | <.001 | <.001* |
|  | Desmosterol | n = 145 | 0.527 + 0.194  (n = 55) | 0.833 + 0.262  (n =90) | <.001 | <.001* |
|  | Sitosterol | n = 135 | 2.22 + 1.16  (n = 53) | 2.70 + 1.74  (n = 82) | .261 | .380 |
| HDL | 7DHC | n = 60 | 0.134 + 0.102  (n = 49) | 0.129 + 0.087  (n = 11) | .901 | .901 |
|  | Lathosterol | n = 86 | 0.713 + 0.754  (n = 70) | 0.572 + 0.410  (n = 16) | .617 | .659 |
|  | Desmosterol | n = 60 | 0.583 + 0.224  (n = 49) | 0.584 + 0.367  (n = 11) | .411 | .479 |
|  | Sitosterol | n = 59 | 2.19 + 1.21  (n = 48) | 2.58 + 1.44  (n = 11) | .419 | .479 |
| ApoA1 | 7DHC | n = 60 | 0.131 + 0.111  (n = 31) | 0.136 + 0.085  (n = 29) | .290 | .386 |
|  | Lathosterol | n = 86 | 0.656 + 0.838  (n = 44) | 0.719+ 0.534  (n = 42) | .096 | .171 |
|  | Desmosterol | n = 60 | 0.509 + 0.206  (n = 31) | 0.662 + 0.276  (n = 29) | .023 | .052* |
|  | Sitosterol | n = 59 | 1.93 + 1.20  (n = 31) | 2.62 + 1.24  (n = 28) | .016 | .044* |
| ApoB | 7DHC | n = 60 | 0.090 + 0.060  (n = 25) | 0.164 + 0.110  (n = 35) | .002 | .006* |
|  | Lathosterol | n = 86 | 0.481 + 0.272  (n = 36) | 0.835 + 0.866  (n = 50) | .026 | .052* |
|  | Desmosterol | n = 60 | 0.437 + 0.142  (n = 25) | 0.687 + 0.264  (n = 35) | <.001 | <.001* |
|  | Sitosterol | n = 59 | 1.96 + 0.99  (n = 25) | 2.48 + 1.39  (n = 34) | .184 | .295 |

† All analyses had a BHC of 16; * Indicates statistical significance.

a AGRE, Autism Genetic Research Exchange; ApoA1, apolipoprotein A1; ApoB, apolipoprotein B; ASD, autism spectrum disorder; BHC, Benjamini-Hochberg p value correction; Cent, centile; CHL, cholesterol; HDL, high-density lipoprotein cholesterol; SD, standard deviation; 7DHC, 7-dehydrocholesterol.

# Supplementary Table 16: Kruskal Wallis analyses of <5th and >5^th^ centile groups of cholesterol, HDL, ApoA1 and ApoB with head circumference, height, weight and BMI in AGRE subjects.

| Lipid (Grouping variable) | Physical Parameter | | Mean + SD (Lipid <5thCent group) | Mean + SD (Lipid >5thCent group) | Kruskal Wallis p value | Significance p value after BHC† |
| --- | --- | --- | --- | --- | --- | --- |
|  | Head OFC  Centile | n = 149 | 71.9 + 26.1  (n = 63) | 71.5 + 26.8  (n = 86) | .924 | .969 |
|  | Height Centile | n = 84 | 57.4 + 30.3  (n = 34) | 69.5 + 31.8  (n = 50) | .043 | .231 |
| Cholesterol |  |  |  |  |  |  |
|  | Weight Centile | n = 91 | 61.1 + 35.2  (n = 38) | 74.1 + 28.4  (n = 53) | .080 | .231 |
|  | BMI (kg/m2) | n = 65 | 18.6 + 4.57  (n = 25) | 21.6 + 6.86  (n = 40) | .089 | .231 |
|  | Head OFC  Centile | n = 76 | 72.0 + 27.2  (n = 60) | 74.0 + 22.7  (n = 16) | .969 | .969 |
| HDL | Height Centile | n = 43 | 54.6 + 33.7  (n = 32) | 73.7 + 29.5  (n = 11) | .072 | .231 |
|  | Weight Centile | n = 43 | 60.7 + 36.5  (n = 36) | 80.3 + 21.6  (n = 7) | .308 | .410 |
|  | BMI (kg/m2) | n = 30 | 20.1 + 6.30 | 20.6 + 3.99 | .378 | .432 |
|  |  |  | (n = 24) | (n = 6) |  |  |
|  | Head OFC  Centile | n = 76 | 76.7 + 25.5  (n = 33) | 69.1 + 26.5  (n = 43) | .117 | .233 |
|  | Height | n = 43 | 54.5 + 31.3 | 62.1 + 34.8 | .290 | .410 |
| ApoA1 | Centile |  | (n = 15) | (n = 28) |  |  |
|  |  |  |  |  |  |  |
|  | Weight  Centile | n = 43 | 57.4 + 39.3  (n = 19) | 69.1+ 31.3  (n = 24) | .293 | .410 |
|  | BMI (kg/m2) | n = 30 | 19.3 + 6.52 | 20.6 + 5.67 | .288 | .410 |
|  |  |  | (n = 9) | (n = 21) |  |  |
|  | Head OFC  Centile | n = 76 | 75.4 + 23.7  (n = 29) | 70.5 + 27.6  (n = 47) | .372 | .432 |
| ApoB | Height Centile | n = 43 | 49.5 + 27.3  (n = 17) | 66.0 + 35.9  (n = 26) | .101 | .231 |
|  |  |  |  |  |  |  |
|  | Weight  Centile | n = 43 | 55.4 + 35.9  (n = 18) | 70.1+ 33.9  (n = 25) | .087 | .231 |
|  | BMI (kg/m2) | n = 30 | 17.2 + 3.39  (n = 12) | 22.2 + 6.39  (n = 18) | .006 | .095‡ |

† All analyses had a BHC of 16; ‡ Indicates trend towards statistical significance.

a AGRE, Autism Genetic Research Exchange; ApoA1, apolipoprotein A1; ApoB, apolipoprotein; BHC, Benjamini-Hochberg p value correction; BMI, body mass index; Cent, centile; HDL, high-density lipoprotein cholesterol; OFC, occipital frontal circumference; SD, standard deviation.

# Supplementary Table 17: Kruskal Wallis analyses of <5^th^ and >5^th^ centile groups of CHL, HDL, ApoA1 and ApoB with Vineland and IQ in AGRE subjects.

| Lipid (Grouping variable) | Measure | | Standard Score Mean + SD (Lipid  <5thCent  group) | Standard Score Mean + SD (Lipid  >5thCent  group) | Kruskal Wallis p value | Significance p value after BHC† |
| --- | --- | --- | --- | --- | --- | --- |
| CHL | Vineland Adaptive | n = 404 | 59.1 + 24.9 | 62.8 + 18.9 | .160 | .310 |
|  | Behavior Composite |  | (n = 78) | (n = 326) |  |  |
|  | Vineland Adaptive | n = 286 | 55.3 + 22.6 | 68.8 + 15.6 | <.001 | <.001* |
|  | Behavior Composite |  | (n = 68) | (n = 218) |  |  |
|  | Vineland Communication | n = 286 | 62.7 + 26.9 | 74.8 + 18.9 | .002 | .003* |
|  | Domain |  | (n = 68) | (n = 218) |  |  |
| HDL | Vineland Daily Living Skills Domain | n = 286 | 55.9 + 25.4  (n = 68) | 69.5 + 17.1  (n = 218) | <.001 | .001* |
|  | Vineland Socialization | n = 286 | 55.3 + 20.9 | 66.9 + 14.3 | <.001 | <.001* |
|  | Domain |  | (n = 68) | (n = 218) |  |  |
|  | Vineland Motor Skills | n = 268 | 82.9 + 21.6 | 89.6 + 18.8 | .050 | .066‡ |
|  | Domain |  | (n = 58) | (n = 210) |  |  |
|  | Vineland Adaptive | n = 286 | 53.6 + 22.1 | 67.4 + 17.1 | .001 | .003* |
|  | Behavior Composite |  | (n = 37) | (n = 249) |  |  |
|  | Vineland Communication Domain | n = 286 | 62.8 + 28.6  (n = 37) | 73.2 + 20.1  (n = 249) | .071 | .081‡ |
| ApoA1 | Vineland Daily Living Skills | n = 286 | 53.8 + 24.6 | 68.1 + 18.8 | .002 | .003* |
|  | Domain |  | (n = 37) | (n = 249) |  |  |
|  | Vineland Socialization | n = 286 | 53.5 + 18.3 | 65.7 + 16.1 | <.001 | .001* |
|  | Domain |  | (n = 37) | (n = 249) |  |  |
|  | Vineland Motor Skills | n = 268 | 84.0 + 20.7 | 88.7 + 19.4 | .216 | .216 |
|  | Domain |  | (n = 32) | (n = 236) |  |  |
| ApoB | Vineland Adaptive | n = 286 | 61.3 + 23.2 | 66.6 + 17.0 | .194 | .310 |
|  | Behavior Composite |  | (n = 56) | (n = 230) |  |  |
| CHL | Full Scale IQ | n = 177 | 92.8 + 23.8 | 91.7 + 26.4 | .975 | .975 |
|  |  |  | (n = 22) | (n = 155) |  |  |
| HDL | Full Scale IQ | n = 150 | 78.5 + 37.1 | 93.5 + 23.6 | .237 | .316 |
|  |  |  | (n = 12) | (n = 138) |  |  |
| ApoA1 | Full Scale IQ | n = 150 | 92.8 + 20.0  (n = 9) | 92.3 + 25.5  (n = 141) | .537 | .614 |
| ApoB | Full Scale IQ | n = 150 | 84.7 + 27.6 | 93.6 + 24.6 | .075 | .199 |
|  |  |  | (n = 22) | (n = 128) |  |  |

† All analyses had a BHC of 8 (initially for 4 lipids analyzed with the Vineland ABC and FSIQ, and then for the HDL and ApoA1 levels analyzed with the 4 Vineland subscales).

* Indicates statistical significance. ‡ Indicates trend toward statistical significance.

a AGRE, Autism Genetic Research Exchange; ApoA1, apolipoprotein A1; ApoB, apolipoprotein B; BHC, Benjamini-Hochberg p value correction; Cent, centile; CHL, cholesterol; HDL, high-density lipoprotein cholesterol; IQ, intelligence quotient; SD, standard deviation; Vineland ABC, Vineland Adaptive Behavior Composite.

# Supplementary Table 18: Kruskal Wallis analyses of <5^th^ and >5^th^ centile groups of HDL and ApoA1 with Vineland in 37 AGRE subjects who had 7DHC measured.

| Lipid (Grouping variable) | Measure | | Standard Score Mean + SD (Lipid <5thCent  group) | Standard Score Mean + SD (Lipid  >5thCent group) | Kruskal Wallis p value | Significance p value after BHC† |
| --- | --- | --- | --- | --- | --- | --- |
| HDL | Vineland Adaptive Behavior Composite | n = 37 | 51.6 + 26.7  (n = 28) | 70.7 + 20.1  (n = 9) | .082 | .082‡ |
| ApoA1 | Vineland Adaptive Behavior Composite | n = 37 | 46.8+ 23.8  (n = 16) | 63.4 + 26.4  (n = 21) | .080 | .082‡ |

† The analyses had a BHC of 2 (for the 2 lipids analyzed with Vineland ABC).

‡ Indicates trend towards statistical significance.

a ApoA1, apolipoprotein A1; BHC, Benjamini-Hochberg p value correction; Cent, centile; HDL, high-density lipoprotein cholesterol; SD, standard deviation; Vineland ABC; Vineland Adaptive Behavior Composite.

**Supplementary Table 19: Classification of ApoA1 and ApoB into patterns in 367 independent AGRE subjects**

| 62 (17 percent) subjects had apolipoprotein A1 (ApoA1) levels<5thCent  81 (22 percent) subjects had apolipoprotein B (ApoB) levels <5thCent  34 (9 percent) had both ApoA1 and ApoB levels <5thCent (HABL)  28 (8 percent) had ApoA1 levels <5thCent + ApoB levels ≥ 5thCent (HAL)  47 (13 percent) had ApoB levels <5thCent + ApoA1 levels ≥ 5thCent (HBL)  258 (70 percent) had both ApoA1 and ApoB levels ≥ 5thCent (Normal) | | | | | | | | | |
| --- | --- | --- | --- | --- | --- | --- | --- | --- | --- |
| Apolipoprotein Categories HABL, HAL, HBL, Normal | Age in years Mean + SD | CHL  Mean + SD  (mg/dL) | CHL%  Mean + SD (mg/d)[†] | HDL  Mean + SD  (mg/dL) | HDL%  Mean + SD | ApoA1 Mean + SD  (mg/dL) | ApoA1% Mean + SD | ApoB Mean + SD  (mg/dL) | ApoB% Mean + SD |
| HABL  (n = 34)  M 28 , F 6 | 7.7 + 3.9 | 77.56  + 25.95  [3] † | 2.53 + 8.14 | 18.88 + 10.42  [2] † | 1.66 + 3.34 | 83.49 + 24.20  [3] † | 1.14 + 1.44 | 41.21 + 9.81  [6] † | 0.93 + 1.34 |
| HAL  (n = 28)  M 23, F 5 | 9.5 + 5.5 | 113.21  + 36.90  [0] † | 9.19 + 22.64 | 21.75 + 7.69  [0] † | 1.26 + 2.28 | 96.92 + 12.48  [0] † | 1.86 + 1.69 | 79.63 + 20.52  [0] † | 44.73 + 33.16 |
| HBL  (n = 47)  M 35, F 12 | 8.1 + 4.2 | 117.55  + 31.57  [0] † | 12.69 + 23.53 | 42.21 + 17.56  [0] † | 32.13 + 32.17 | 137.77 + 23.36  [0] † | 50.34 + 30.82 | 45.96 + 9.61  [4] † | 1.48 + 1.58 |
| Normal  (n = 258)  M 219, F 39 | 9.3 + 5.1 | 161.57  + 37.25  [3] † | 46.18 + 33.38 | 46.17 + 16.90  [4] † | 41.04 + 33.19 | 152.39 + 27.25  [0] † | 66.45 + 29.10 | 85.28 + 25.66  [0] † | 54.07 + 33.04 |

[ ] † Indicates the number of subjects with a result below the floor of the lipid assay and assigned the floor value (CHL 50 mg/dL, HDL 3 mg/dL, ApoA1 19 mg/dL, ApoB 26 mg/dL)

a AGRE, Autism Genetic Research Exchange; Apo, apolipoprotein; ApoA1, apolipoprotein A1; ApoB, apolipoprotein B; Cent, centile; HDL, high-density-lipoprotein-cholesterol; SD, standard deviation; %, Centile.

# Supplementary Table 20: Kruskal Wallis analyses of the 3 apolipoprotein patterns of HABL, HAL, HBL with Vineland and IQ in AGRE subjects.

| Apo Disorder (Grouping variable) | Measure | | Standard Score Mean + SD  (In Apo group) | Standard Score Mean + SD (Not in Apo group) | Kruskal Wallis p value | Significance p value after BHC† |
| --- | --- | --- | --- | --- | --- | --- |
|  | Vineland Adaptive Behavior  Composite | n = 286 | 50.5 + 23.4  (n = 22) | 66.9 + 17.4  (n = 264) | .003 | .017* |
| HABL | Vineland  Communication Domain | n = 286 | 58.5+ 28.0  (n = 22) | 73.0 + 20.7  (n = 264) | .033 | .044* |
|  |  |  |  |  |  |  |
| (Hypo ApoA1  + Hypo ApoB) vs. all other subjects | Vineland Daily  Living Skills Domain | n = 286 | 49.6 + 25.5  (n = 22) | 67.7 + 19.1  (n = 264) | .002 | .003* |
|  |  |  |  |  |  |  |
|  | Vineland  Socialization Domain | n = 286 | 51.2 + 19.4  (n = 22) | 65.2 + 16.2  (n = 264) | .002 | .003* |
|  | Vineland Motor Skills Domain | n = 268 | 86.6 + 17.3  (n = 18) | 88.3 + 19.8  (n = 250) | .654 | .654 |
| HAL (Hypo ApoA1 with ApoB that is hyper or normal)  vs. all other subjects | Vineland Adaptive Behavior Composite | n = 286 | 58.0 + 20.1  (n = 15) | 66.0 + 18.3  (n = 271) | .137 | .277 |
| HBL (Hypo ApoB with ApoA1 that is normal or hyper)  vs. all other subjects | Vineland Adaptive Behavior Composite | n = 286 | 68.3 + 20.5  (n = 34) | 65.2 + 18.1  (n = 252) | .383 | .460 |
| HABL |  |  |  |  |  |  |
| (Hypo ApoA1  + Hypo ApoB) | Full Scale IQ | n = 150 | 90.1 + 17.4  (n = 7) | 92.4 + 25.5  (n = 143) | .378 | .460 |
| vs. all other subjects |  |  |  |  |  |  |
| HAL (Hypo ApoA1 |  |  |  |  |  |  |
| with ApoB that is  hyper or normal) | Full Scale IQ | n = 150 | 102.0 + 33.9  (n = 2) | 92.2 + 25.1  (n = 148) | .731 | .731 |
| vs. all other subjects |  |  |  |  |  |  |
| HBL (Hypo ApoB |  |  |  |  |  |  |
| with ApoA1 that is  normal or hyper) | Full Scale IQ | n = 150 | 82.2 + 31.5  (n = 15) | 93.5 + 24.2  (n = 135) | .138 | .277 |
| vs. all other subjects |  |  |  |  |  |  |

† The initial analysis had a BHC of 6 (for the 3 apolipoprotein patterns analyzed with the Vineland ABC and FSIQ), and then a BHC of 4 (for HABL analyzed with the 4 Vineland subscales);

* Indicates statistical significance.

a AGRE, Autism Genetic Research Exchange; Apo, apolipoprotein; ApoA1, apolipoprotein A1; ApoB, apolipoprotein B; BHC, Benjamini-Hochberg p value correction; FSIQ, full scale intelligence quotient; IQ, intelligence quotient; SD, standard deviation; Vineland ABC, Vineland Adaptive Behavior Composite.
