# Supplementary material for: Sterol and lipid analyses identifies hypolipidemia and apolipoprotein disorders in autism associated with adaptive functioning deficits
Source: Transl Psychiatry. 2021 Sep 9;11:471. doi: 10.1038/s41398-021-01580-8 (PMC8429516; doi:10.1038/s41398-021-01580-8)
Supplement: Supplementary file 2 — Index of Supplementary Tables 1 to 20_8–12–2021 [file 41398_2021_1580_MOESM2_ESM.docx]

**Index of Supplementary Tables 1 to 20:**

Supplementary Table 01, NHANES-III age and sex specific cholesterol percentile values 4.0-16.9 years (Supp_Table_01_NHANES-III_percentiles_cholesterol_4.0-16.9 yrs_Tierney_Transl_Psychiatry_2021)

Supplementary Table 02, NHANES-III age and sex specific HDL-cholesterol percentile values 4.0-16.9 years (Supp_Table_02_NHANES-III_percentiles_HDL_4.0-16.9 yrs_Tierney_Transl_Psychiatry_2021)

Supplementary Table 03, NHANES-III age and sex specific ApoA1 percentile values 4.0-16.9 years (Supp_Table_03_NHANES-III_percentiles_ApoA1_4.0-16.9 yrs_Tierney_Transl_Psychiatry_2021)

Supplementary Table 04, NHANES-III age and sex specific ApoB percentile values 4.0-16.9 years (Supp_Table_04_NHANES-III_percentiles_ApoB_4.0-16.9 yrs_Tierney_Transl_Psychiatry_2021)

Supplementary Table 05, NHANES-III age and sex specific calculated LDL-cholesterol percentile values 4.0-16.9 years (Supp_Table_05_NHANES-III_percentiles_calculated_LDL_4.0-16.9 yrs_Tierney_Transl_Psychiatry_2021)

Supplementary Table 06, NHANES-III age and sex specific triglyceride percentile values 4.0-16.9 years (Supp_Table_06_NHANES-III_percentiles_ triglycerides_4.0-16.9 _Tierney_Transl_Psychiatry_2021)

Supplementary Table 07, NHANES-III age and sex specific cholesterol values 17.0-90.0 years

(Supp_Table_07_NHANES-III_percentiles_chol_17.0-90.0 yrs_Tierney_Transl_Psychiatry_2021)

Supplementary Table 08, NHANES-III age and sex specific HDL-cholesterol percentile values 17.0-90.0 years (Supp_Table_08_NHANES-III_percentiles_HDL_17.0-90_yrs_Tierney_Transl_Psychiatry_2021)

Supplementary Table 09, NHANES-III age and sex specific ApoA1 percentile values 17.0-90.0 years

(Supp_Table_09_NHANES-III_percentiles_ApoA1_17.0-90.0 yrs_Tierney_Transl_Psychiatry_2021)

Supplementary Table 10, NHANES-III age and sex specific ApoB percentile values 17.0-90.0 years (Supp_Table_10_NHANES-III_percentiles_ApoB_17.0 years-90.0 yrs_Tierney_Transl_Psychiatry_2021)

Supplementary Table 11, NHANES-III age and sex specific calculated LDL-cholesterol percentile values 17.0-90 years (Supp_Table_11_NHANES-III_percentiles_calculated_LDL_17.0-90.0 yrs_Tierney_Transl_Psychiatry_2021)

Supplementary Table 12, NHANES-III age and sex specific triglyceride percentile values 17.0-90.0 years (Supp_Table_12_NHANES-III_percentiles_triglycerides_17.0-90.0 yrs_Tierney_Transl_Psychiatry_2021)

Supplementary Table 13: AGRE subjects with low and high levels of cholesterol, HDL, ApoA1, and ApoB.

Supplementary Table 14: AGRE subject cholesterol and lipoprotein characterization: subject number, <5th and >5th centile groups’ mean levels, age, assay information.

Supplementary Table 15: Kruskal Wallis analyses of <5th and >5th centile groups of cholesterol, HDL, ApoA1 and ApoB with 7DHC, lathosterol, desmosterol and sitosterol in AGRE subjects.

Supplementary Table 16: Kruskal Wallis analyses of <5th and >5th centile groups of cholesterol, HDL, ApoA1 and ApoB with head circumference, height, weight and BMI in AGRE subjects.

Supplementary Table 17: Kruskal Wallis analyses of <5th and >5th centile groups of CHL, HDL, ApoA1 and ApoB with Vineland and IQ in AGRE subjects.

Supplementary Table 18: Kruskal Wallis analyses of <5th and >5th centile groups of HDL and ApoA1 with Vineland in 37 AGRE subjects who had 7DHC measured.

Supplementary Table 19: Classification of ApoA1 and ApoB into patterns in 367 independent AGRE subjects.

Supplementary Table 20: Kruskal Wallis analyses of the 3 apolipoprotein patterns of HABL, HAL, HBL with Vineland and IQ in AGRE subjects.
